# Influence of the Imagination or the Will upon the Pregnant Woman

**Published:** 1852-07

**Authors:** I. G. Braman

**Affiliations:** Brighton, Mass.


Communicated for the Boston Med. and Surg. Journal.

*Influence of the Imagination or Will upon the Pregnant Woman.* By I. G. Braman, M.D., Brighton, Mass.

The following somewhat unique case occurred in my practice, and is submitted for the pages of the Journal without note or comment.

In the month of May, 18—, I was summoned to attend Mrs. ———, who was at the close of the ninth month of pregnancy. As I entered the room, I found everything arranged for her accouchement, which was momentarily expected to occur. The pains were frequent and vigorous, and an examination par vaginam revealed the os uteri fully dilated, the head advancing, and all things as favorable for a speedy termination as could be desired. I consoled myself with the idea that I should soon be released and on my way home. The female assistants, those kind and *sometimes* convenient appendages to the lying-in room, concurred most fully in this opinion, and were profuse in their encouragement and congratulations to my patient. But alas for the vanity of all earthly expectations. She did not respond either in faith or by practice. On the contrary, she obstinately turned a deaf ear to all consolation, declaring in emphatic terms that "she should not be confined until aunt Nancy came back." By the way, this same aunt Nancy was a woman of considerable note in that portion of the obstetric world, and Mrs. ——— had a special arrangement with her in reference to this occasion, but the *miserable sinner*, regardless of her solemn promise, had left town on a visit. Her presence and sympathy it seemed was a *sine qua non;* and consequently I must relinquish every hope of accomplishing anything, while such an unfortunate conjunction of circumstances obtained. In vain I laughed, expostulated, and even scolded. Mrs. ——— made but one reply to all! "You may say and do what you please, but I tell you I shall not be sick before aunt Nancy comes back, if she never comes." The pains were still urgent, and a few expulsory efforts were all that appeared necessary to complete the labor.

In this state of doubt and uncertainty we spent the night.

Morning came, but with it no relief. The major part of the day was passed in the same manner—matters remained *in statu quo.* About 4, P.M., my assistants (who had received some accession to their number from a neighboring domicile) began to look grave, and exchanged significant glances. Suddenly they vanished, leaving me *solus* with Mrs. ———. By certain stifled whisperings, I inferred they were holding a conference in an adjoining room. This, I knew, portended some important communication to myself, and I waited with fortitude to hear what it might be. I was not kept long in suspense. The door opened, and marshalled in single file, they advanced, when the oldest, who had evidently been chosen chief speaker, thus addressed me :

"Doctor, do you not think Mrs. ——— has been sick sometime?"

"I do."

"Why is she not confined?"

"You have heard what she says, and can judge as well as I."

"Is anything out of the way?"

"No."

"Can't something be done to help her along?"

"I know of nothing. We must wait patiently."

"Are you willing we should try an experiment on her?"

"It depends on what it is."

"We won't do anything to hurt her."

"Well, on such a condition you may try your experiment, but I shall interfere if I see anything in it calculated to do harm."

With this consent they speedily commenced operations. A common wash tub was placed under a chair which had lost the whole or the greater part of its bottom. In this tub some wormwood, hops, and I think tansy, were put, and boiling water poured over them. After waiting a few minutes, for the water to cool a little, Mrs. ——— was taken from her bed, seated in the chair, duly propped up by pillows, and supported by the arms of all the feminine gender present. This process was accompanied with various appropriate remarks, such as—"There, now we have fixed you nicely." "You will be sick right off." "We aint a going to stay here again all night," &c., &c. Contrary, however to their expectations, her pains immediately ceased. She was perfectly comfortable, and evidently enjoyed the change. The conclave stood aghast, and after waiting over an hour, gave up their experiment, and with much chagrin replaced the good woman upon her bed. There she remained one fortnight, happy and contented, suffering no annoyance, except some slight derangement of the stomach, which was easily relieved by appropriate remedies. At the expiration of this period, aunt Nancy fortunately came back. No sooner did Mrs. ——— hear of this than her pains returned. Aunt Nancy was sent for, I was again summoned, and, in a very short space of time, a fine girl made its debut into the world.

